# Exploring perspectives on antimicrobial stewardship: a qualitative study of health managers in Kenya

**DOI:** 10.1186/s41256-020-00177-w

**Published:** 2020-11-17

**Authors:** Samuel Mungai Mbugua, George Njoroge, Caroline Kijogi, Moses Kamita, Rachel Kimani, Peter Mwaura, Bibianne Waiganjo Aidi, Jesse Gitaka

**Affiliations:** Mount Kenya University, Thika, Kenya

**Keywords:** Antimicrobial stewardship, Antimicrobial resistance, Building blocks/low-lying fruits

## Abstract

**Background:**

Antimicrobial resistance is a significant public health concern with the establishment of antimicrobial stewardship in hospitals being increasingly obligatory. Perspectives and insights of health managers on antimicrobial stewardship (AMS), complementary health services and building blocks are imperative towards implementation of robust AMS programs. This study aimed to understand perspectives of hospital managers on AMS and identify areas of management engagement while addressing potential blockades to change.

**Methods:**

A cross-sectional, qualitative, multicenter study was conducted in three hospitals in Kenya. Key-informant interviews on perspectives on AMS were administered to hospital managers. Qualitative data was captured using audio tapes and field notes, transcribed and managed using NVivo 12 software. An iterative process was used to develop the thematic framework and updated in two rounds of iteration analysis. Analysis charts for each emergent theme were developed and categorized across all participants.

**Results:**

Perspectives on AMS are described in five thematic categories; Importance of antimicrobial stewardship and the role of medicines and therapeutics committee, availability of antimicrobial formulary and usage surveillance systems, laboratory competency and recommendations for infection prevention and management, educational resources and communications channels available, building blocks and low-lying fruits for Antimicrobial Stewardship Committees. The role of stewardship collaboration in diagnosis and antimicrobial prescription was alluded to with managers indicating a growing rise in occurrence of antimicrobial resistance. There lacked contextualized, hospital specific antimicrobial formulary and adequate laboratory competency. Staff training and communication channels were available in varying capacity across the three hospitals. Building blocks identified include medicines and therapeutics committee, education, and training platforms (Continuous Medical Education and Continuous Professional Development activities) and hospital leadership commitment.

**Conclusions:**

The practice of antimicrobial stewardship is not implemented and well developed as demonstrated by lack of core AMS complementary health services. However, the health managers are aware of the fundamental importance of antimicrobial stewardship programs and the vast benefits of implementation and institutionalization of AMS to hospitals and their clients. The findings underpin the importance of understanding and incorporating perspectives of health managers on existing contextual mechanisms that can be leveraged on to establish robust AMS programs in the fight against antimicrobial resistance.

## Background

Antimicrobial resistance is a significant public health threat heightened by lack of development of new antimicrobial agents [1, 2]. The correlation between antimicrobial resistance and antimicrobial use is without doubt. Antimicrobial stewardship (AMS) programs exist to ensure and promote judicious appropriate antimicrobial use in hospitals [3]. In Kenya, AMS programs remain underdeveloped coupled with high antibiotic use in hospitals, lack of system-wide monitoring of infection prevention and control measures, and lack of awareness among healthcare workers [4].

Core elements and checklist items for hospital AMS programs have been developed with the objective of clearly defining principles on the basis of effectiveness and affordability of antimicrobials [5]. AMS processes are established in hospitals with introduction of a knowledge base and social structures that sometimes conflict with traditional hierarchies of decision making and consultation [6]. This qualitative study, in part, examines the current existing mechanisms of surveillance and monitoring of the use of antimicrobials in these contextual settings and how they can be used as building blocks in the establishment of AMS. This is important because studies have shown that AMS models focusing exclusively on delivering advice on antimicrobial usage rather than incorporating management of existing interpersonal relationships and mechanisms may limit their capacity in optimizing antimicrobial use [7]. Efforts addressing AMR often target the prescriber’s behavioral change, focusing on optimizing antibiotic prescribing aimed at reducing overuse and inappropriate use [8]. AMS should use a collaborative-persuasive approach of counselling and education to increase clinicians’ ability to prescribe appropriately with awareness of the potential unintended consequences of inappropriate antibiotic usage. Effective implementation of AMS programs will depend on structural, organizational, and cultural contexts identified prior to implementation [9].

Optimization of antimicrobial use in hospitals through stewardship is an increasingly fundamental priority within the context of proliferating antimicrobial resistance [7]. Indeed, emphasis on enforcement of AMS guidelines should be more explicit than ever [10]. Understanding the perspectives of hospital managers to AMS is vital towards the establishment of robust and sustainable antimicrobial stewardship programs and the engagement of management and addressing potential blockades to change. Health managers, referred herein, are defined as the heads of various departments in the hospital responsible for planning, directing and coordination of health workers and healthcare provision across different disciplines, and members of the hospital management committee. This key-informant interview study was conducted to describe the health managers’ perspectives of characteristics and strategies in place (low-lying fruits) that can be maximized in the establishment of AMS programs.

## Methods

### Setting and research design

This was a cross-sectional, qualitative, multicenter study conducted in three hospitals. The levels of service delivery in Kenya are arranged in four tiers; tier 1: community level, tier 2: primary care level, tier 3: county level, tier 4: national level (11). The three facilities fall under tier 3 providing generalized health services; comprehensive medical, surgical, diagnostic and rehabilitative care each with a catchment population of one million people. The hospitals are located in two counties in Kenya serving as county and Sub-county teaching and referral hospitals. Convenience sampling was used to select the three facilities with intended purpose of establishment of AMS programs. The sampled facilities entailed two county referral hospitals (level V facilities according to the Kenya Health Policy 2014–2030) [11] and one Sub-county referral hospital (level IV facility). Due to ethical considerations, two facilities (one county referral hospital and the Sub-county referral hospital) located in one county are designated as county I in this study, and the one county referral hospital in another county referred to as county II. The study was nested within an ongoing antimicrobial stewardship project that levers on implementation science approaches to understanding and enhancing AMS best practices in select health facilities in Kenya [12].

The key informant interview participants were selected purposively based on potential knowledge on AMS and health care delivery possessed. The recruitment strategy involved engaging with members of the hospital management committee during the study sensitization workshops and the participant-led AMS workplan development. Ten participants were invited to participate in the study with two unavailable for the interview due to work-related concerns. The participants were drawn from departmental managers in two county referral hospitals and one Sub-county referral hospital. We interviewed two medical superintendents, three nurse managers, two hospital pharmacists and one Sub-county pharmacist manager. The sample population targeted hospital managers in key departments and members of the hospital management committee (Table 1).

**Table 1:** Characteristics of respondents

Designation	Duration at current position Versus experience	Work setting	Location
Medical superintendent	2 years vs more 5 years’ experience	County referral hospital	County I
	1-year vs more 5 years’ experience	Sub-county referral hospital	
Pharmacist	1-year vs more 2 years’ experience	County referral hospital	County II
	1-year vs more 5 years’ experience	Sub-county referral hospital	County I & II
	2 years vs more 5 years’ experience	Sub-county manager	County II
Nursing services manager	More than 5 years vs more 15 years’ experience	County referral hospital	County I & II
	More than 5 years vs more 15 years’ experience	Sub-county referral hospital	County I

### Data collection

Trained research assistants under the supervision of research scientists collected qualitative data using face-to-face key-informant interviews that were conducted using a semi-structured interview guide. The content of training for research assistants encompassed phenomenological philosophy of research, unstructured nature of key informant interviews and skills in qualitative interviewing. Quality assurance was ensured through prolonged engagement with health managers during the process of qualitative data collection and AMS establishment; reflexivity, confirmability and dependability ensured through use of a field diary and a thorough audit trail of the research study path and activities.

### Data analysis

Qualitative data was captured using audio tapes and field notes, transcribed and managed using QSR NVivo 12 software. The KII transcripts were coded and checked for coding consistency using a thematic framework to classify and organize data into five themes using grounded theory approach. An iterative process was used to develop the thematic framework and updated in two rounds of iteration analysis. Analysis charts for each emergent theme were developed and categorized across all participants.

## Results

Our sample comprised of 8 facility managers in two counties in Kenya. Results are presented in 5 thematic categories; 1) Importance of antimicrobial stewardship and the role of medicines and therapeutics committee; 2) availability of an antimicrobial formulary and usage surveillance systems; 3) Laboratory competency and recommendations for infection prevention and management; 4) Educational resources and communications channels available; 5) Building blocks and low-lying fruits for ASCs.

### Theme 1: importance of antimicrobial stewardship and the role of medicines and therapeutics committee

#### Importance of antimicrobial stewardship

All the hospital managers interviewed signified the importance of antimicrobial stewardship in their hospital settings towards the rational use of antimicrobials.

*“I absolutely think anti-microbial stewardship is important in any hospital setting. Actually, it's something that we've been missing here, For a while. Yes, but we have come up with a committee. So in the next few weeks/months and hopefully, we hope we can have it up and running. Yeah. Yeah. But yes, I agree. Yeah”.* [Sub-county I hospital Pharmacist]

*“I think anti-microbial stewardship is key to any hospital, and especially if you create/if you develop resistance to the majority of the common antibiotics, you find that you cannot sustain the high end or new model antibiotics that are coming to the market. So judicious use of antibiotics is key to any institution.”* [County hospital I Medical Superintendent]

The role of stewardship collaboration in diagnosis and antimicrobial prescription was alluded to with managers indicating a growing rise in occurrence of antimicrobial resistance as highlighted by a Sub-county pharmacist and hospital nurse manager;

*“I think it should be a primary objective and I’m also very interested in it because where we are going, we have seen a lot of resistance, a lot of resistant bugs especially when you're working in ICU settings, bugs that were usually not a big problem and becomes an issue. So I think this is something that needs to be dealt with. I'm happy to see the efforts being made by the county government and the national government towards that.”* [Sub-county II pharmacist]

*“…… Yes it should be one of management’s objectives. One it’s because we have so many patients taking these antibiotics’, and with the taking of antibiotics we don’t know which one is good for the patient or which one is not good for the patient because we don’t have a way in which we check.”* [County hospital II nursing services manager]

#### Role of the medicines and therapeutics committee

Prior to this study, the function of regulation of judicious use of antimicrobials was under the mandate of the medicines and therapeutics committees in the various hospitals. The key functions of these committees identified by the managers include leadership in management of antimicrobial utilization and infection prevention and control.

i. Function 1: Leadership in management of antimicrobial utilization

*“So the medicines and therapeutics committee has been handling everything to do with medicine in this hospital.”* [Sub-county Hospital I Pharmacist]

*“Ideally, it's the committee that should be dealing with everything. Therapeutics and antimicrobial stewardship are now inculcated as some of the functions of the Medicines and Therapeutics Committee {MTC}.”* [County hospital II Pharmacist]

*“Okay. We have an MTC which is chaired by our pharmacist. That one is like an oversight committee, which sees how the utilization of these antibiotics is done in the hospital and also it assists in identifying and purchasing of the antibiotics generally in the facility because most of the time we buy drugs with the hospital revenue. Most of the time.* [Sub-county hospital I Nursing Services Manager]

ii. Function 2: Infection prevention and control

*“What is happening, what is mainly being championed by the MTC, what we mainly have right now, the latest drive, is hand washing.”* [Sub-county II pharmacist]

### Theme 2: availability of an antimicrobial formulary and usage surveillance systems

#### Availability of an antimicrobial formulary

Most hospital and sub county managers indicated that there was no contextualized, hospital specific antimicrobial formulary based on the local antibiograms.

*“What we rely on is the central one. Remember there is a formula or guideline on how to use antibiotics in terms of what is our first line? And what is our second line? So that one is there., but we don't have a local hospital formulary for anti-microbials. But we are trying to come up based on some local statistics and based on some guidelines in terms of evidence based to try and come up with our own.* [County hospital I Medical Superintendent]

*“Not really clear because it may be there with the pharmacist, but it may not be clear to all of the staff because sometimes you may order and then you are told to follow a certain regimen. Yeah, but it may not be very clear to all the staff as such, maybe we just need to streamline that and bring it up clearly.* [County hospital I Nursing Services Manager]

Only one respondent mentioned that the hospital had an antimicrobial formulary based on the essential medicines lists. This facility is also the only one that had made steps towards establishment of an antimicrobial stewardship committee;

*“The hospital has the hospital formulary, so that they focus on oral antibiotics, Antiprotozoas, there is another section on parental antibiotics but they are part of the hospital formulary.”* [Sub-county hospital I pharmacist]

*“Is not based on the new guidelines but I think they met two weeks ago and this is the MTC, and the idea was to update the formulary based on the essential medicines lists.* [Sub-county hospital I Medical Superintendent]

#### Availability of an antimicrobial use surveillance system

The critical role of monitoring the usage of antimicrobials is mainly under the pharmacy department in most of the hospitals according to two pharmacists’ respondents.

*“Yeah, so we follow that in-house…. Patient by patient. For example, if a patient's been brought on ceftriaxone and it becomes an extended cost where you suspect that they're probably treating meningitis or something like that, then we will try to follow up and see that it is actually what they are treating. The system is mainly in house and it is done in the pharmacy and it is on a patient to patient basis here.”* [Sub-county hospital I Pharmacist]

*“Majorly, this hospital relies on the clinical pharmacist but also the pharmacists in the departments and dispensing areas especially the inpatient dispensing areas... even our technicians are aware. They give us calls….”* [Sub-county II pharmacist]

However, there is a dire need for the establishment of antibiotic usage monitoring mechanisms in the three facilities in this study as highlighted by the hospital managers.

*“This is not a place (antimicrobial use surveillance system) within our system*. “[Sub-county Hospital I Medical Superintendent]

*“It hasn't started yet. But in the committee, we had mentioned it. I think when the committee really picks up it will be one of the issues to look at.”* [County hospital I Nursing Services Manager]

*“We don't really have quite an established monitoring system as far as antimicrobial use is concerned.”* [County hospital II Pharmacist]

In line with antimicrobial use surveillance and monitoring, hospital managers emphasized on the imperative role of clinicians in ensuring judicious use of antimicrobials.

*“So the clinicians are the ones who make most of these diagnoses that are bacterial infections that require antibiotics to be prescribed. At the moment, the laboratory has some way of doing antimicrobial sensitivity. If we can get a structure where the pharmacy is getting the right antibiotics and stock and dispensing according to properly prescribed procedures, and then clinicians are following a strict code of prescribing antibiotics and culture and sensitivity where its required and the laboratory is, of course, carrying out a culture and sensitivity, to the largest extent, they can.”* [Sub-county hospital I pharmacist]

A respondent felt that the clinicians in the facility did not practice judicious use of antimicrobials.

*“….I think it is not appropriate, because most of the time many patients go with different antibiotics, today different antibiotics, tomorrow, different antibiotics, you know the sequence of using them is not right.”* [County hospital II Nursing Services Manager]

The nurse managers provided a key insight that would be critical in establishment of antimicrobial utilization surveillance and monitoring systems. One nurse manager reflected on the role played by medical representatives in influencing the prescription patterns of clinicians.

*“currently, from my own observation, we have so many medical representatives and when they come to the facility, they come and introduce their drugs secretly to the prescribers with some incentives to them. So you find that the prescribers really prescribe what is brought by the medical representatives. Yeah... because they have been given some incentives, they want to sell, not really what the patient will benefit from and the cost maybe very high.”* [County Hospital I Nursing Services Manager]

### Theme 3: laboratory competency and recommendations for infection prevention and management

#### Laboratory competency

All hospital managers indicated a need for laboratory strengthening, mainly around laboratory infrastructure and availability of ideal laboratory reagents and equipment. Culture and sensitivity testing are a main concern with patients relying on services from private facilities.

*“I believe that the biggest gap in the laboratory has been in terms of culture and sensitivity, because when you get those stubborn bugs and you have to take samples and send them to XYZ it would be easier if they were just done here then treatment will be started quicker. Yeah. So if they can get their culture and sensitivity up to date, then I think that would be a huge step.”* [Sub-county I hospital Pharmacist]

*“Unfortunately, we are at zero, as far as saying that it is an infection or not we use full hemogram, maybe that, but specifically doing cultures so that you can tell which organisms we have not, we are actually going to that or we actually have to send patients outside*.” [County hospital II pharmacist]

There were also views from respondents that clinicians were not keen on utilizing laboratory services available indicating a need for sensitization on appropriate laboratory testing to mitigate against antimicrobial resistance.

*“So I want to believe the issue of cultural and sensitivity, It's something that even the laboratory has been keen in sensitizing the users like clinicians to be ordering more of the cultures and sensitivity tests which as it is now, there has been slow utilization. This is one area where we need to , as the management, advocate for more.”* [Sub-county hospital I Medical Superintendent]

*“For us the lab is able to do Cultural and sensitivity, within our facility although the uptake has not been that good because of the marketing of services. And also probably maybe turnaround time, but we are trying to see if we can market their services to the customers in terms of the clinicians so that they can take up the use of culture and sensitivity which will inform their antibiotics.”* [County hospital I Medical Superintendent]

Two of the hospitals’ laboratory were ISO certified with one respondent indicating that the facility’s laboratory had highly competent personnel.

*“. And then actually we met the levels of ISO certification. We have specialized lab technologists, the highest qualified person there has a masters. Yeah. Others are BSc. Holders. Others have diplomas….but higher national diplomas. So compared with other laboratories around here, we have the highest qualified personnel.”* [Sub-county hospital I Nursing Services Manager]

#### Recommendations for infection prevention and management

The respondents presented varying scenarios in regards to infection prevention and management. The consensus was that all three facilities relied on national guidelines on infection, prevention and control.

*“We have some guidelines. I saw that there's a guideline from the WHO but we don't have that. But on national guidelines, we have a few guidelines.”* [Sub-county hospital I Pharmacist]

*“What we rely on is the national guideline, we don’t have local guidelines. The national guidelines, I think are updated from time to time. So that's what we have been relying on. But we don’t have any locally.”* [County hospital I Medical Superintendent]

*“Infection Prevention and Control, We have guidelines again, developed by the IPC and the national policy guideline. “*[County hospital II Nursing Services Manager]

### Theme 4: educational resources and communications channels available

#### Educational resources

The hospital managers indicated that continuous medical education was the main education resource utilized in staff training to optimize antimicrobial prescription and stewardship.

*“….we do have Continuous Medical Education (CME) and some training, especially being a training facility. I know this one of the areas where our institutions normally focus on… evidence-based medicine. And I think it goes best hand-in-hand with knowing what works in the set up and I think we really need to have it more organized so that everyone in the hospital is actively involved and participates.”* [Sub-County hospital I Medical Superintendent]

*“Continuous Professional Development (CPDs), Our professional organizations give us a lot if CMEs on that especially Pharmaceutical Society of Kenya, the Hospital Pharmacists Association of Kenya… think that’s our most important source of educational resources.* [Sub-county II Pharmacist]

One respondent however noted that there haven’t been any staff trainings conducted especially in regard to antimicrobial resistance.

*“The gap is there but what is not there is that I don’t know why people have not been able to do any CMEs. Because we have not been talking anything about antimicrobials.”* [County hospital II Nursing Services Manager]

#### Communication channels

One way of communication of audits/reviews on the quality/appropriateness of antimicrobial use is through the Medicines and Therapeutics Committee via memos and meetings as indicated by one pharmacist.

*“We tried to communicate through the MTC, we didn’t follow up know whether it reached the people on the ground but since we are still working together, we are still able to incorporate that information in our daily ward rounds.”* [Sub-county II pharmacist]

The other channel communication utilized is through the CME trainings with managers mentioning a need for strengthening CMEs to achieve dissemination of information.

*“We at times go through our CMEs, we have a very vibrant CME. But I think I’d want to say we normally pass information but at times we miss out on many of the staff. So that’s why I said earlier on we need to have a structured way of communicating such issues. Because like even though its in place, its not …the information does not go through well to reach everybody.* [Sub-county hospital I Medical Superintendent]

*“and what we do is that each department, each medical department usually have their own CME meetings. And on those days, we have feedback, usually from surgical team, gynecological team or pediatrics team or the medical team, We normally give the feedback and the action is responded at, just especially considering we have interns, traffic of interns, so when we noticed problems, especially as far as antibiotic use is concerned.”* [County hospital II Pharmacist]

### Theme 5: building blocks and low-lying fruits for ASCs

The integral building blocks and low-lying fruits that hospital managers identified towards the formation and institutionalization of robust antimicrobial stewardship committees include using the MTCs in the formation of ASCs, laboratory strengthening, and using CMEs to sensitize clinicians and nurses on various aspects of antimicrobial resistance and stewardship.

*“Yeah. I think what we can really rely on MTC to help to initiate this antimicrobial stewardship, is the MTC.”* [County Hospital I Nursing Services Manager]

*“I think the low-lying fruits that we already we have started harvesting, one is formation of the Medicine Therapeutics Committee is one fruit that we have already harvested, and we are trying to come up with one. Number two, probably from that, we need to form some other splinter committee to do for us antimicrobial stewardship. And then we need to strengthen our lab in terms of maybe investing more to ensure that to do culture and sensitivity and maybe shorten the turnaround time”* [County hospital I Medical Superintendent]

Financial support during the process of establishment was mentioned as building block.

*“….One, the most important thing for us is, is having defined systems that anybody can use, even if a doctor is not there. There is, for example, having scheduled meeting or evaluation exercises that need to be done or, for example, communication or established system is one of them..Second, financial support is really important. Yeah. for it work. And another thing is to educate people on the importance of it. Be it consultants be it nurses, I mean all carders, for me, those are the three things that I feel that need to be there for it to actually function.”* [County hospital II pharmacist]

## Discussion

The study aimed to understand the perspectives of hospital managers to AMS and identify areas of management engagement and addressing potential blockades that need to be changed. In consensus with several studies globally [13, 14], hospital managers opined to the importance of antimicrobial stewardship with emphasis on the role of stewardship collaboration in diagnosis and antimicrobial prescription. In a multi-country study on the cultural and contextual determinants of antimicrobial stewardship programs across low, middle- and high-income countries, Charani et al., [15] concluded that there is need for promotion of interdisciplinary team work depending on available health workforce in AMS. AMS programs should incorporate the behavioral change wheel [16, 17] based on the Theoretical Domains Framework [18] whilst incorporating the cultural and contextual drivers of antimicrobial decision making [19]. Our findings therefore are in congruence with global research literature on the importance of cognizance of perceptions, gaps, challenges, and potential building blocks in the establishment of functional AMS programs.

Hospital managers emphasized the importance of antimicrobial formulary and communication on antimicrobial audits to health workers through the medicines and therapeutics committee and CME trainings. Relevant workshops, CMEs and conferences could play a critical role towards improving future antibiotic prescribing to overcome the threat of antimicrobial resistance [20]. Hayat et al. [21] and Gebreteckle et al. [22] resonate with this finding stating that specific priorities like formulary restriction and hospital-wide audits with feedback on antimicrobial usage, accompanied with regular educational resources, are key strategies in implementation of AMS programs in Pakistan. In answering the question of multi-professional education support for better stewardship, Pereira et al. [23] identified four key messages; (i) re-design of health professional education system within an organization is required for stewardship education; (ii) delivery of simplified messages that are relevant to the professional and clinical context as core success of interventions; (iii) developing competencies that are relevant and suitable is vital before implementing educational solutions (alluding to staff’s professional competency); (iv) a core requirement of the educational process should be the measurement of engagement with educational resources. Cherani et al. [24] identifies gaps in adequate training and education of health workers participating in stewardship while emphasizing the need for greater involvement of pharmacists and nurses for effective and sustainable AMS programs.

This study highlights the need for laboratory strengthening in terms of infrastructure and availability of reagents and equipment in the participating facilities. This correlates with Hayat et al. [21] findings on contribution of absence of standard diagnostic facilities towards the irrational prescription of antibiotics. Black et al. [25] stated lack of resources as a barrier to improvement of use of antimicrobials.

A vital group of respondents in this study were the nurse managers in the three hospitals. In these hospitals, antimicrobials are administered by nurses with the perceptions from participating nurse managers offering invaluable insights across all five themes identified. Efforts should be tailored towards ensuring participation and engagement of nurses in AMS, addressing their knowledge needs and the context of their work [26]. Nurse-driven antimicrobial stewardship is perceived as an extension of the role of the nurse as a patient’s advocate and is critical to accelerating patient safety initiatives in antimicrobial management [26, 27].

The study identified three key low-lying fruits/building blocks that can be capitalized on in the establishment of AMS programs; the existing medicines and therapeutics committees, strengthening of laboratory competency and use of CMEs to train and disseminate key messages on antimicrobial resistance and stewardship strategies. An additional low-lying fruit is financial support and commitment from the national government and county health leadership. An institution with limited resources can use Infection Prevention Control (IPC) committees to build on antimicrobial stewardship. Hospital staff with roles in IPC can take additional roles in AMS due to human resource constraints in developing countries. The members of IPC also need adequate training on AMS.

However, some limitations to our study should be noted. This study was conducted in three hospitals and cannot depict an overall picture of the perceptions of all health managers and, in reflection, all hospitals in Kenya. Having stated this, the study findings do provide generalizable knowledge on the contextual factors and strategies existent and applicable in public hospitals in Kenya that can help in the establishment of AMS programs. Only the perceptions of managers in public hospitals were sought and the views of health managers in private hospitals may differ from those expressed in this study coupled with differences in organizational structures.

## Conclusion

The practice of antimicrobial stewardship is not implemented and well developed in the sampled healthcare facilities. The facilities have critical deficits in core AMS complementary health services such as adequate, essential laboratory services and antimicrobial usage surveillance and monitoring systems. Nonetheless, the health managers resonate an understanding of the fundamental importance of antimicrobial stewardship programs and the vast benefits of implementation and institutionalization of AMS to the hospital and its clients alike. The AMS building blocks and low-lying fruits identified in this qualitative study will be instrumental in the establishment and implementation of AMS programs. These include the medicines and therapeutics committee, education and training platforms (CMEs and CPDs) and hospital leadership commitment towards antimicrobial stewardship. Health managers are critical entities in any AMS program. Their perspectives are significant in understanding existing contextual mechanisms that can be leveraged on to establish robust AMS programs in the fight against AMR. The study findings underpin the importance of understanding and incorporating the perspectives of health managers on the existing contextual mechanisms that can be leveraged on to establish robust Antimicrobial Stewardship programs in the fight against antimicrobial resistance. The findings also emphasize the need to create hospital leadership buy-in in order to overcome challenges in AMS programs in the response to antimicrobial resistance.

## Data Availability

The datasets used and/or analyzed during this study are available from the corresponding author on reasonable request.

## Abbreviations

AMS: Antimicrobial stewardship
AMR: Antimicrobial Resistance
CME: Continuous Medical Education
CPD: Continuous Professional Development
MTC: Medicines and Therapeutics Committee
ASC: Antimicrobial stewardship Committee
